# Supplementary material for: Epigenetic Modulation and Neuroprotective Effects of Neurofabine-C in a Transgenic Model of Alzheimer’s Disease
Source: Genes (Basel). 2025 Oct 15;16(10):1214. doi: 10.3390/genes16101214 (PMC12562421; doi:10.3390/genes16101214)
Supplement: Supplementary file 1 [file genes-16-01214-s001.zip › genes-3861314-supplementary.pdf]

SUPPLEMENTARY DATA

Table S1: Neurofabine-C composition.

Cocoa extract	*Theobroma cacao*
Faba bean extract	*Vicia faba L.*

COCOA (*Theobroma cacao*)

References: Edo G I, Samuel P O, Oloni G O, et al. Review on the Biological and Bioactive components of Cocoa (*Theobroma Cacao*). Insight on Food, Health and Nutrition. *Natural Resources for Human Health*. **2023**, *3*:426-448. doi:10.53365/nrfhh/174302.

Table S2: The nutritional content of cocoa in percentage per 100g.

**Macronutrients**	**Percentage**
Protein	15-21%
Carbohydrates	~15%
Lipids	10-25%
Fiber	25-40%
**Micronutrients**	**Per 100g**
Vitamin A(Retinol)	<0.2mg
Vitamin E(Tocopherol)	2.5mg
Vitamin B1(Thiamine)	0.3mg
Vitamin B1(Riboflavin)	0.4mg
Vitamin B3(Niacin)	0.7mg
**MINERALS**	**Per 100g**
Sodium (Na)	0.03g
Potassium (k)	4.3g
Calcium (ca)	151mg
Phosphorus (P)	700mg
Iron (Fe)	26mg
Magnesium (mg)	555mg

Macronutrients	Percentage
Copper (cu)	5mg.

Bioactive Compounds of Cocoa

Phenolics

Among the many bioactive substances included in cocoa are phenolics, which include procyanidins, flavonoids, and catechins. These phenolic chemicals contribute significantly to the antioxidant qualities of cocoa in addition to giving it its unique flavor and scent (Melo et al., 2021). Because of its antioxidant properties, cocoa phenolics assist the body fight off dangerous free radicals, which lessens oxidative stress and may lower the chance of developing a number of chronic illnesses, including cancer and heart problems. Moreover, Sorrenti and colleagues (2020) have linked these phenolic compounds to anti-inflammatory properties.

Phenolic Composition Analysis of Cocoa Varieties

Cocoa beans naturally contain phenolic compounds, such as flavonoids and polyphenols, which are beneficial substances. According to Martin et al. (2021), they are crucial in determining the nutritional value and sensory attributes of cocoa and chocolate. According to Urbańska and Kowalska (2019), these chemicals have an impact on the flavor profile as well as the antioxidant characteristics that have drawn attention to cocoa and dark chocolate due to their possible health advantages. This section examines the phenolic makeup of many cocoa cultivars, each distinguished by a particular flavor profile and set of characteristics. By conducting a thorough examination and measurement of these phenolic chemicals in cocoa mucilage and cocoa bean shell, we hope to gain important understanding of the distinctions and parallels between these types.

Table S3: Compounds found in Cocoa and their formulas, structures and functions.

S/N	COMPOUND	FORMULA	FUNCTIONS/USES	
1	flavanols	C15H14O2	They have anti-inflammatory, cardioprotective, anti-cancer, hypolipidemic, anti-diabetic, anti-microbial, and anticancer properties.	(Urbańska & Kowalska, 2019)
2	Anthocyanidins	C15H11O+	I. They mostly serve the purpose of coloring plant parts with red, blue, and purple shades, among other colors. ii. They help to avoid diabetes and obesity. III.Anthocyanins have been proved to have the ability to modify cognitive and motor performance, improve memory, and play a part in delaying the effects of aging on cerebral function.	(Jimmy & Jose, 2011).
3	Proanthocyanidins	C31H28O12	I. They boost blood vessel strength in those who have diabetes (increased blood sugar level) and high blood pressure (hypertension). ii. They helps in the prevention of cancer iii. They also assist in the treatment of nutritional deficiencies.	(Liang et al., 2021)

S/N	COMPOUND	FORMULA	FUNCTIONS/USES	
4	Caffeine	$C_8H_{10}N_4O_2$	It helps the brain and neurological system to be more active. it also causes a greater flow of hormones like cortisol and adrenaline throughout the body. Caffeine can also help you feel awake and alert in moderation.	(Pucciarelli, 2013)
5	Theobromine	$C_7H_8N_4O_2$	It functions as a diuretic, a vasodilator, and a cardiac stimulant. Additionally, it might help in the treatment of fatigue and orthostatic hypotension.	(Feo et al., 2020)
6	Methylxanthines	$C_6H_6N_4O_2$	They relax the smooth muscles of the bronchi, esophagus, and gastroesophageal sphincter.	(Ludovici et al., 2017)
7	Catechin	$C_{15}H_{14}O_6$	They assist control vascular tone by triggering endothelial nitric oxide. Due to the anti-inflammatory effects of the capillaries and the suppression of vascular cell growth factors involved in atherosclerosis, it also contributes significantly to the prevention of the progression of atherosclerosis.	(Mendoza-Meneses, Feregrino-Pérez, & Gutiérrez-Antonio, 2021)
8	Procyanidins	$C_{30}H_{26}O_{13}$	They protect the circulatory and heart systems. They might act as antioxidants and prevent nitrosamine formation. They might guard against their effects in healthy cells. Together, they and vitamin C reduce the risk of breast cancer.	(Balentić et al., 2018)

Bibliography:

- Melo TS, Pires TC, Engelmann JVP, Monteiro ALO, Maciel LF, Bispo EDS. Evaluation of the content of bioactive compounds in cocoa beans during the fermentation process. J Food Sci Technol. 2021 May;58(5):1947-1957.
- Sorrenti V, Ali S, Mancin L, Davinelli S, Paoli A, Scapagnini G. Cocoa Polyphenols and Gut Microbiota Interplay: Bioavailability, Prebiotic Effect, and Impact on Human Health. Nutrients. 2020 Jun 27;12(7):1908.
- Martin MÁ, Ramos S. Impact of cocoa flavanols on human health. Food Chem Toxicol. 2021 May;151:112121.
- Urbańska B, Kowalska J. Comparison of the Total Polyphenol Content and Antioxidant Activity of Chocolate Obtained from Roasted and Unroasted Cocoa Beans from Different Regions of the World. Antioxidants (Basel). 2019 Aug 6;8(8):283.

FABA BEAN (*Vicia faba L.*)

References:

- Dhull, S. B., Kidwai, M. K., Noor, R.,Chawla, P., & Rose, P. K. A review of nutritional profile and processing of faba bean (*Vicia faba L.*). *Legume Science*. **2022**, *4*:e129.
- Martineau-Côté D, Achouri A, Karboune S, L'Hocine L. Faba Bean: An Untapped Source of Quality Plant Proteins and Bioactives. *Nutrients*. **2022,** *14*:1541.

Table S4: Nutritional composition of raw and cooked faba beans (per 100 g).

**Composition**	**Unit**	**Immature seeds**		**Mature seeds**	
		**Raw[b]**	**Cooked[b] (with salt)**	**Raw[b]**	**Cooked[c] (no salt)**
Proximate:					
Water	g	81	83.7	10.98	--
Energy	kcal/kj	72/301	62/259	341/1425	110/460
Protein	g	5.6	4.8	26.12	7.6
Total lipid (fat)	g	0.6	0.5	1.53	0.4
Ash	g	1.1	0.9	3.08	--
Carbohydrate, by difference	g	11.7	10.1	58.29	19.6
Fiber, total dietary	g	4.2	--	25	5.4
Minerals:					
Calcium	mg	22	18	103	36.0
Iron	mg	1.9	1.5	6.7	1.5
Magnesium	mg	38	31	192	43.0
Phosphorus	mg	95	73	421	124.7
Potassium	mg	250	193	1,062	268.2
Sodium	mg	50	277	13	5.0
Zinc	mg	0.58	0.47	3.14	1.0

Composition	Unit	Immature seeds		Mature seeds	
		Raw[b]	Cooked[b] (with salt)	Raw[b]	Cooked[c] (no salt)
Vitamins[a]:					
Vitamin C, total ascorbic acid	mg	33	19.8	1.4	0.3
Niacin	mg	1.5	1.2	2.832	0.7
Folate, total	µg	96	58	423	104.1
Vitamin A	IU	350	270	53	15.0
Vitamin K (phylloquinone)	µg	nr	nr	9	2.9

- [a] Thiamin, riboflavin, pantothenic acid, and vitamin B6: all <1.0 mg in raw and cooked faba beans.
- [b] *Source*: USDA (2021).
- [c] *Source*: Nutrition Data (2021).

Table S5: Amino acid content of immature and mature faba beans.

Amino acid	Immature seeds	Mature seeds
Tryptophan	0.056	0.247
Threonine	0.208	0.928
Isoleucine	0.251	1.053
Leucine	0.432	1.964
Lysine	0.366	1.671
Methionine	0.043	0.213
Cystine	0.077	0.334
Phenylalanine	0.228	1.103
Tyrosine	0.196	0.827
Valine	0.274	1.161

Amino acid	Immature seeds	Mature seeds
Arginine	0.463	2.411
Histidine	0.134	0.664
Alanine	0.228	1.07
Aspartic acid	0.631	2.916
Glutamic acid	0.855	4.437
Glycine	0.23	1.095
Proline	0.252	1.099
Serine	0.246	1.195

- *Source*: USDA (2021).

RAW DATA FROM BIOCHEMICAL ANALYSIS

Neurofabine-C ID	GROUP	WBC	RBC	HGB	HCT	MCV	MCH	MCHC	PLT	MPV	PDW	PCT	VB6 serum	VB9 plasma	VB12 serum	ALB serum	TAS serum	MDA_ plasma	Aβ42 serum	Aβ42_NC (pg/mg protein)	Aβ42_HIP (pg/mg protein)
DT22	1	0.79	9.89	14.5	49.4	49.9	14.7	29.4	899	5.6	14.7	0.503	6.88	30.25	8394	**4.8**	2.06	8.74	36.76	105.85	241.95
DT23	1	2.56	9.83	14.7	51.1	52.0	15.0	28.8	1161	5.8	14.6	0.673	7.86	13.24	20040	3.9	1.29	3.46	40.06	104.72	182.04
DT24	1	1.25	9.66	14.2	48.5	50.2	14.7	29.3	687	5.6	14.8	0.385	17.96	15.05	10780	4.9	2.63	11.65	43.66	ND	ND
DT25	1	0.96	9.07	13.5	45.9	50.6	14.9	29.4	1077	5.7	14.5	0.614	8.54	12.67	11580	3.3	1.66	4.70	79.46	ND	ND
DT26	1	2.52	9.71	14.9	52.3	53.9	15.3	28.5	839	5.6	14.6	0.470	11.39	15.61	9445	3.3	1.34	6.59	88.84	120.96	187.05
DC27	0	1.39	8.66	12.9	44.9	51.9	14.9	28.7	886	5.3	14.4	0.470	11.23	18.44	ND	3.1	1.35	4.24	51.02	138.61	171.86
DC28	0	2.9	9.68	15.0	51.5	53.2	15.5	29.1	921	5.4	14.6	0.497	19.67	24.35	11723	4.2	0.63	2.36	49.22	168.76	230.64
DC29	0	1.94	10.32	15.4	53.9	52.2	14.9	28.6	806	5.5	14.6	0.443	10.58	27.53	13144	4.1	0.38	3.19	50.06	143.13	213.26

RAW DATA FROM EPIGENETIC ANALYSIS

Neurofabine-C ID	GROUP	FOXO3	ATM	HIF1α	TRP73	APOE	HDAC3	DNMT3a	5mc
DT22	1	3.47	19.7	0.22	18.2	0.24	0.4	9.1	0.03576115; 0.02805118
DT23	1	2.4	8.34	0.36	31.6	0.37	0.31	23.1	0.02739501; 0.02427822
DT24	1	1.65	5.9	0.35	24.3	0.61	0.47	18.1	0.01383071; 0.01574803

DT25	1	3.99	11.92	0.15	29.6	0.53	0.51	19.8	0.03139764; 0.04458661
DT26	1	0.75	13.32	0.23	17.5	0.72	0.39	15.7	0.03464567: 0.02588583
DC27	0	1	1	1	1	1	1	1	0.00556102; 0.00127953
DC28	0	0.8	1.1	0.81	5.5	1.02	0.76	1.09	0.00378937; 0.00733268
DC29	0	0.56	1.06	1.24	3.8	0.85	1.28	1.03	0.00816929; 0.00521654
